# Direct deposition of silver nanoplates on quartz surface by sequence pre-treatment hydroxylation and silanisation

**DOI:** 10.1016/j.mex.2017.11.008

**Published:** 2017-11-15

**Authors:** Norhayati Abu Bakar, Muhamad Mat Salleh, Akrajas Ali Umar, Joseph George Shapter

**Affiliations:** a Institute of Microengineering and Nanoelectronics (IMEN), Universiti Kebangsaan Malaysia, 43600, Bangi, Selangor, Malaysia; b Flinders University, School of Chemical and Physical Sciences, Bedford Park, Adelaide, South Australia, 5001, Australia

**Keywords:** Direct deposition of colloidal silver nanoplates on quartz surface using a self–assembly technique, Adhesion, Deposition, High density, Hydroxylation, Silanisation, Silver nanoplates film

## Abstract

Silver nanoparticles deposited on quartz substrates are widely used as SERS substrates. The nanoparticles can be deposited directly from colloidal solution by dipping technique. However, the adhesion of the particles on the quartz surface is very poor. Normally the substrate is pre-treated with hydroxylation or silanisation process. In this paper, we have demonstrated that the application of the sequence pre-treatment hydroxylation and silanisation have improved the density of silver nanoplates desposited on the quartz surface.

• Sequence hydroxylation and silanisation pre-treatment assists the deposition of the nanoplate on the surface.
• Various immersion times of the quartz surface into the colloidal nanoplates determined size distributions and density surface of the nanoplates on the surface.

## Graphical abstract

**Figure:**
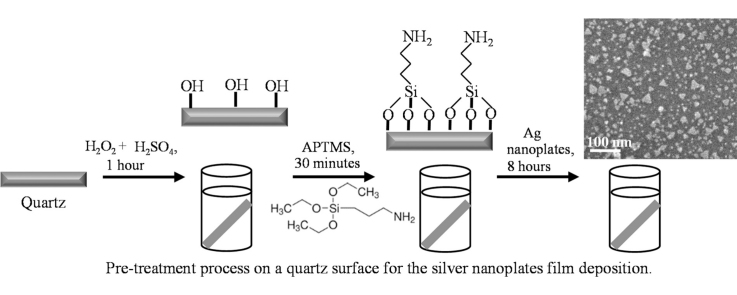

## Method details

The present study was designed to deposit triangular silver nanoplates thin film on a quartz surface from the colloidal nanoplates using a self-assembly technique. Triangular-shaped silver nanoplates were synthesised as reported previously using direct chemical reduction approach [1]. The materials used were quartz substrate (Latech Scientific Supply, Kuala Lumpur, Malaysia), 3-aminopropyltrimethoxysilane 97% (Sigma-Aldrich, Kuala Lumpur, Malaysia), sulphuric acid 95% (Sigma-Aldrich), 30% hydrogen peroxide (HmbG Chemicals, Kuala Lumpur, Malaysia) and ethanol absolute (HmbG Chemicals). The normal-grade quartz substrate without a special specification was used with a measurement of 1.2 × 1.2 cm. These chemicals were used as received without any further purification.

Silver nanoplates thin film was prepared using a self-assembly technique by immersing a cleaned quartz surface into the colloidal nanoplates. In order to obtain a high density of the nanoplates on the surface, effects of pre-treatment on the quartz surface were studied prior to being immersed into the colloidal. After that, the various immersion times of the quartz surface into the colloidal nanoplates was carried out to observe the coverage of nanoplates distribution on the surface.

## Pre-treatments on a cleaned quartz substrate surface

**Fig. 1:**
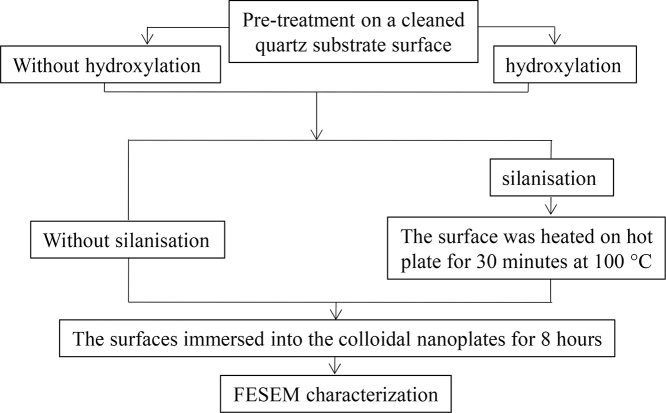
A flow chart of the pre-treatments on the quartz surface to prepare the nanoplate thin film.

Two pre-treatment studies, namely hydroxylation and silanisation were done on a cleaned quartz surface to study adhesion of the nanoplates on the surface. Quartz substrate was consecutively cleaned in acetone and then 2-propanol using a sonication process for 15 min. After that, the surface was dried and prepared for pre-treatment prior to depositing the nanoplates on the surface by immersing the surface into the colloidal nanoplates. Flow chart for pre-treatment preparation on the surface is described in Fig. 1.

In a typical pre-treatment process, the cleaned quartz substrates were first treated with and without hydroxylation treatment. Hydroxylation treatment was prepared by immersing the cleaned quartz surface for 1 h in 1:3 (v/v) hydrogen peroxide:sulphuric acid. After that, the surface was taken out and consecutively cleaned in deionised water and then ethanol using a sonication process for 15 min. Finally, the surface was rinsed with a copious amount of deionised water and then was dried in air.

These untreated and treated surfaces with hydroxylation were then carried out by being treated with and without silanisation treatment. Silanisation treatment involved immersing the surfaces into a 3-aminopropyltrimethoxysilane (APTMS) solution with 5% concentration at room temperature for 1 h. The 5% concentration of APTMS solution was prepared by diluting 5 mL of 97% APTMS in an absolute ethanol. The selected type of solvent is able to provide a huge impact on density and APTMS film formation on the surface. Therefore, an anhydrous solvent is used as APTMS solvent to obtain a uniform monolayer deposition [2]. The formed APTMS films were rinsed with copious ethanol and then were dried in air. After that, the APTMS films were heated on a hot plate for 30 min at 100 °C.

An ultimate products of the two pre-treatments on the quartz surfaces were labelled as described in Table 1. These samples were then immersed into the colloidal nanoplates for 8 h. After that, the formed nanoplate films were rinsed with copious amounts of deionised water and then were dried in air for field emission scanning electron microscopy characterisation.

**Table 1:** Four samples of the quartz surface after pre-treatment processing on the surface.

Samples	Hydroxylation treatment	Silanisation treatment
S1	x	x
S2	x	√
S3	√	x
S4	√	√

**Fig. 2:**
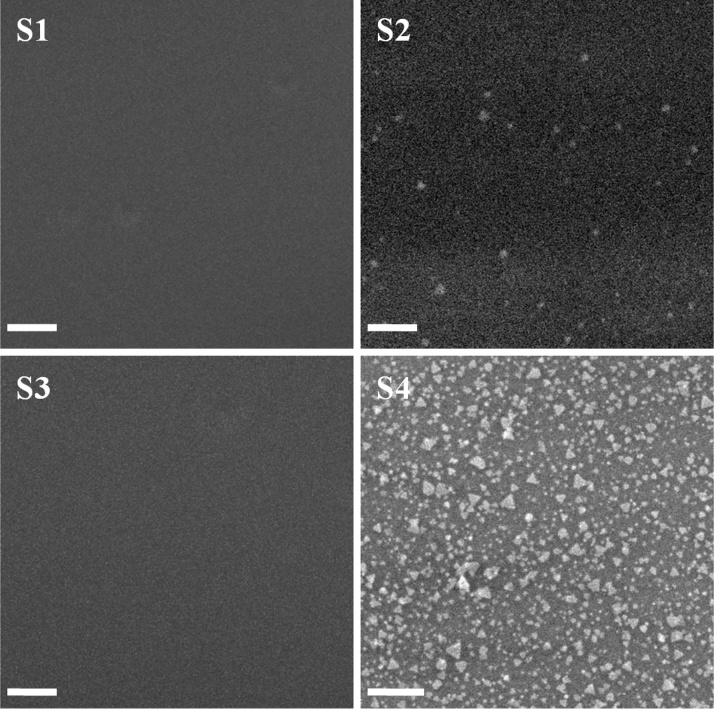
Field emission scanning electron microscopy images of the nanoplate films on the untreated and treated quartz surfaces. Scales are 100 nm.

An attachment of the nanoplate particles on the S1 to S4 surfaces was observed using field emission scanning electron microscopy images, as shown in Fig. 2. Based on the results, the nanoplates were not successfully attached on the untreated quartz surface, S1, or on the hydroxylation-treated surface, S3, after immersion for 8 h into the colloidal nanoplates. For the sinalisation-treated surface, S2 has revealed the nanoplate particles randomly attached on the surface with a small distribution. However, two sequence pre-treatments on the quartz surface assisted a good adhesion of the nanoplates on the surface, as shown on the S4 image. The triangular nanoplate particles were abundantly distributed and formed a monolayer nanoplate film on the quartz surface. A high coverage was obtained after the surface was treated by hydroxylation and then silanisation treatments prior to immersion into the colloidal nanoplates for 8 h.

The following is a brief explanation about two pre-treatment processes that result in a good deposition of silver nanoplates on the quartz surface. In this pre-treatment, hydroxylation treatment that used a mixture of sulphuric acid and hydrogen peroxide would fill the quartz surface site with polar hydroxyl molecule, OH^−^. Therefore, this hydroxylation treatment changed a hydrophobic surface property to hydrophilic [3], which is easy to dampen by water. After that, the surfaces were washed using an ultrasonic process and were then treated with silanisation treatment. The quartz surface site was then replaced with an aminosilane molecule after the hydroxyl molecule on the surface site attracted the aminosilane molecule from the APTMS solution. This aminosilane molecule was positioned at each surface site of the quartz, and the surface developed higher hydrophilic properties [4]. Additionally, the aminosilane molecule could promote the adhesion of nanoplate particles on the quartz surface due to the strong interaction between the amine group and the metal nanoparticles [5], [6]. This might be the reason for the existence of a small distribution of the nanoplate deposition on the S2 surface, which was treated by sinalisation treatment only. Without hydroxylation treatment, the aminosilane molecule was unable to fill the quartz surface site without being attracted to the surface and then replacing the hydroxyl molecule on the surface.

### Effect of immersion time

The subsequent study is to determine the effect of immersion time into the colloidal nanoplates towards a film deposition. In this deposition process, an optimum pre-treatment surface, S4, was immersed into the colloidal nanoplates at 30 min, 1 h, 2 h, 8 h, 14 h, and 22 h. The formed nanoplate films were then rinsed with a copious amount of deionised water and were dried in air.

**Fig. 3:**
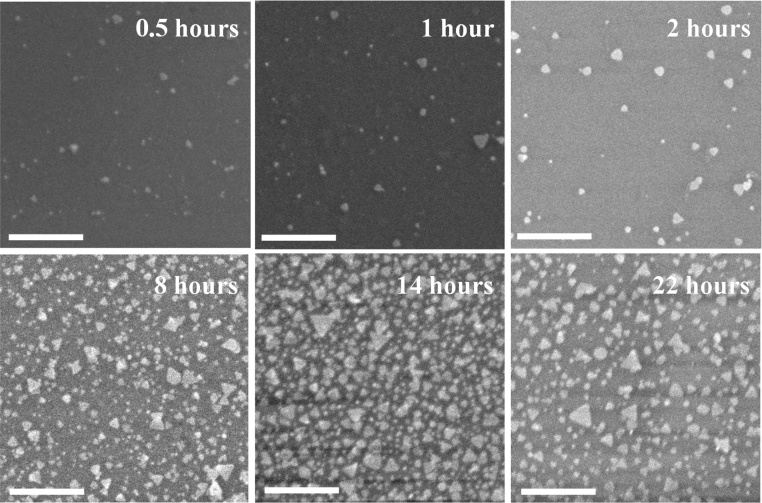
Field emission scanning electron microscopy images of the nanoplate films with six different immersion times. Scales are 100 nm.

**Fig. 4:**
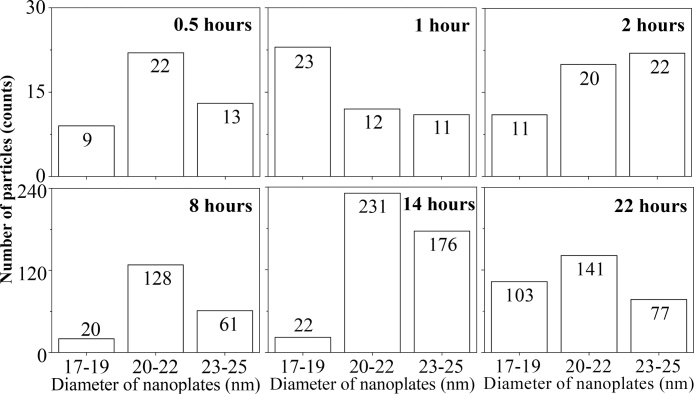
Bar chart of surface density and size distribution of nanoplates on the surface for six different immersion times.

Fig. 3 shows the different coverage of the nanoplates on the surface for different immersion times, labelled as 0.5 h, 1 h, 2 h, 8 h, 14 h and 22 h for 30 min, 1 h, 2 h, 8 h, 14 h and 22 h of immersion time, respectively. The surface density and size distribution of the nanoplates on the surface were measured using image J software. Then, the analysis results of the density and size are plotted for six different immersion time as illustrated in Fig. 4. Referring to the FESEM images, the triangular nanoplate particles were successfully attached on the quartz surface after immersion for 30 min into the colloidal nanoplates. The 0.5-h image revealed that the nanoplate deposition on the surface has a small percentage of distribution with the calculated surface density is 44 nanoplates/μm as shown in Fig. 4. These nanoplates were distributed on the surface without overlapping or agglomerating with other nanoplates, where the average size of the triangular nanoplates is 23.43 ± 1.14 nm.

For longer immersion times, a triangular shape of the nanoplates is still maintained and the diameter of the triangular nanoplates did not change significantly, from 22 to 25 nm. The calculated average size of the nanoplates for the 1-h, 2-h, 8-h, 14-h, and 22-h samples are 22.26 ± 1.90 nm, 24.03 ± 0.76 nm, 24.83 ± 1.70 nm, 24.37 ± 2.87 nm, and 23.98 ± 1.50 nm, respectively. However, the surface density of the nanoplates on the surface increases with the increase of the immersion times, except for 22 h. The average surface densities for the 1-h, 2-h, 8-h, 14-h, and 22-h samples are 46, 53, 209, 429, and 321 nanoplates/μm, respectively. A decrease in the surface density for 22 h of immersion time might be due to the deposited nanoplates on the surface becoming redissolved into the colloidal for a longer immersion time.
